# The association between remnant cholesterol and bone mineral density in US adults: the National Health and Nutrition Examination Survey (NHANES) 2013–2018

**DOI:** 10.1186/s12944-024-02145-6

**Published:** 2024-05-18

**Authors:** Peilun Xiao, Zhihang Wang, Zeyao Lu, Shijia Liu, Chongjun Huang, Ying Xu, Ye Tian

**Affiliations:** 1 Department of Orthopedics, Shengjing Hospital of China Medical University, Shenyang, China; 2 Department of Anesthesiology, Shengjing Hospital of China Medical University, Shenyang, China

**Keywords:** Bone mineral density, Remnant cholesterol, Americans, NHANES

## Abstract

**Background:**

Previous evidence showed a possible link of dyslipidemia with bone health. Nevertheless, the correlation of remnant cholesterol (RC) with bone mineral density (BMD) has yet to be well investigated. This study investigated the association of RC with total spine BMD in general Americans.

**Methods:**

This study explored the relationship of RC with total spine BMD in subjects aged ≥ 20 years from the National Health and Nutrition Examination Survey (NHANES) 2013–2018. After adjusting for covariates, multivariate linear regression and stratified analyses were conducted to determine the correlation of serum RC with total spine BMD in adult Americans. Restricted cubic spline (RCS) was applied to examine the nonlinear association of serum RC with total spine BMD.

**Results:**

This study included 3815 individuals ≥ 20 years old, 1905 (49.93%) of whom were men and 1910 (50.07%) of whom were women. After adjusting for all covariates, the results showed a negative relationship of serum RC with total spine BMD (β= -0.024, 95% CI: -0.039, -0.010). The interaction tests of age, sex, race, and BMI showed no statistically significant effects on the association. The RCS also indicated a negative linear correlation of serum RC with total spine BMD (nonlinear *P* = 0.068, overall *P* < 0.001). Moreover, RC had a stronger effect on total spine BMD than total cholesterol (TC), low-density lipoprotein cholesterol (LDL-C), and high-density lipoprotein cholesterol (HDL-C).

**Conclusions:**

This study found that serum RC was negatively related to total spine BMD in U.S. adults. These findings emphasized the important role of RC in bone health in American adults.

**Supplementary Information:**

The online version contains supplementary material available at 10.1186/s12944-024-02145-6.

## Introduction

Osteoporosis, a common skeletal disorder in elderly individuals, is featured by low bone mineral density (BMD) and high fragility fracture risk [1]. A recent study indicated that 19.7% of the world’s population older than 50 years is suffering from osteoporosis [2]. As the population ages globally, this number still continues [3]. The increasing prevalence of osteoporosis is leading to high morbidity and mortality in elderly people around the world, placing significant health and economic burdens on society [3]. The development of osteoporosis can be attributed to two factors. The first aspect is the peak bone mass (PBM) accumulation during puberty, and the second is the bone mass loss in old age [4, 5]. Bone mass loss can be determined by various factors, including genetics, environment, diet, and others [5, 6]. Therefore, to reduce the high morbidity and mortality caused by osteoporosis in elderly individuals, it is essential to recognize and manage modifiable factors that contribute to osteoporosis.

Dyslipidemia is commonly observed in patients with osteoporosis and is thought to affect BMD by impairing circulation [7–9]. In a study of 4323 Koreans, Kim et al. [7] reported that serum triglyceride levels were negatively related to BMD in men aged > 50 years and postmenopausal women. Cao et al. [8] indicated that total cholesterol (TC) was negatively related to total BMD in U.S. young adults. Although several studies have demonstrated that triglyceride, low-density lipoprotein cholesterol (LDL-C), and TC are negatively correlated with BMD, the findings are not always consistent [10–12]. These inconsistent results limit the clinical utility of lipid biomarkers. There is an urgent need to explore new lipid parameters to predict osteoporosis and intervene.

Residual cholesterol (RC), the cholesterol in triglyceride-rich lipoproteins, is associated with various diseases, including metabolic, cardiovascular, and other disorders [13–15]. RC was shown to be an equal or even more reliable cardiovascular disease predictor than LDL-C or TC [16]. Although the role of RC in cardiovascular disease has been widely investigated, few studies have investigated the role of RC in bone health. An early study suggested that a reduction in the uptake of triglyceride-rich lipoproteins by osteoblasts leads to increased bone formation in mice [17]. In a northern Chinese cohort of 7,053 participants, Hou et al. [18] found that serum RC was negatively associated with BMD in men younger than 60 years after adjustment for a range of confounding factors. Therefore, RC may serve as a new marker of lipid metabolism to influence bone metabolism and BMD.

However, the relationship of RC with BMD has not been assessed in women or older people. In addition, no study has explored their relationship in Americans. Thus, this study aimed to assess the correlation of serum RC with total spine BMD in Americans aged ≥ 20 years from the National Health and Nutrition Examination Survey (NHANES) 2013–2018. We hypothesized that the serum RC is negatively related to total spine BMD.

## Methods

### Study design

**Fig. 1:**
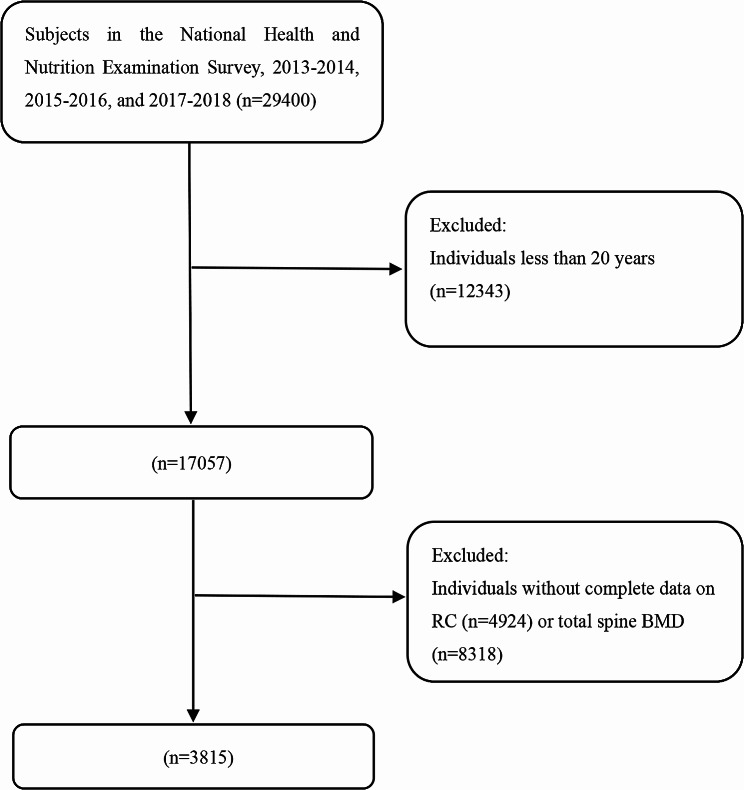
Flowchart of participants selection. BMD: bone mineral density. RC: remnant cholesterol

The NHANES is a national nutrition and health program that collects and publicly releases data biennially in the United States. In detail, the National Center for Health Statistics Ethics Review Committee provided ethical consent for the NHANES program. Each participant or their guardians signed informed consent forms for the NHANES programs. The latest three NHANES cycles were combined: 2013–2014, 2015–2016, and 2017–2018. Individuals aged 20 years or older were enrolled in this study. Initially, 29,400 individuals were enrolled in the NHANES 2013–2018. Then, 12,343 subjects were excluded because they were younger than 20 years, 8318 participants were excluded because of missing information on lumbar spine BMD, and 4924 participants were excluded because of missing RC information. Finally, 3815 individuals were enrolled in this study. The participant selection flowchart is displayed in Fig. 1.

### Assessment of RC and BMD

RC was used as the exposure variable in this study. Peripheral blood was drawn from the subjects for analysis after they fasted for at least eight hours in the morning. Serum triglyceride and TC levels were analyzed using the enzymatic method. Serum High-density lipoprotein cholesterol (HDL-C) was assessed by heparin-manganese precipitation or a direct immunoassay technique method. Serum LDL-C was measured by the Friedwald calculation. Serum RC was determined using the formula [RC = TC- LDL-C - HDL-C], which was in accordance with previous research [19, 20].

The total spine BMD was assessed by dual-energy X-ray absorptiometry (DXA) using Hologic densitometers. Professionals collected and standardized BMD data. Total spine BMD was used as an outcome variable in this analysis. Detailed information on BMD can be found in the DXXLSBMD datasets on the NHANES website [21].

### Covariates

Covariates were chosen based on the published study [22]. Variables such as race, age, sex, body mass index (BMI), blood urea nitrogen, total protein, serum calcium, serum vitamin D, serum phosphorus, serum uric acid, poverty income ratio (PIR), education level, smoking and drinking behaviors, high pressure, diabetes, physical activities, and statin use were considered in the study. The PIR is a widely used indicator of family income. BMI was determined by dividing weight (in kilograms) by the square of height (in metres). Races were divided into five groups (Other Hispanic, Non-Hispanic Black, Mexican American, Other Race, and Non-Hispanic White). Education level was categorized into three categories (College degree or above, High school graduate, and Under high school). Total protein, blood urea nitrogen, serum total calcium, serum phosphorus, serum uric acid, and serum vitamin D were collected via laboratory measurements. Smoking and drinking behaviors, high pressure, diabetes, physical activities, and statin use were determined by questionnaires: Smoke at least 100 cigarettes in life? Have you ever had 5 or more drinks every day? Doctor told you had high blood diabetes? Ever told you had high blood pressure? How many days did you do moderate recreational activities in a typical week? Have you taken statins in the past 30 days? Detailed data on covariates can be seen at the NHANES website.

### Statistical analysis

The present study used the mean ± standard deviation and percentages to represent continuous and categorical variables, respectively. The characteristics of participants were described based on serum RC quartile (Q1: ≤ 0.308 mmol/L, Q2: 0.309–0.464 mmol/L, Q3:0.465–0.699 mmol/L, Q4: ≥ 0.700 mmol/L). To compare differences between the characteristics of participants, the study used weighted linear regression models and weighted χ^2^ tests for continuous variables and categorical variables, respectively. Furthermore, multivariate linear regression analyses were performed to explore the relationship of RC with total spine BMD. Model 1 was built as an unadjusted model. Then, Model 2 was created by adjusting age, race, and sex. Finally, Model 3 was created by adjusting all variables of race, age, sex, BMI, blood urea nitrogen, total protein, serum calcium, serum vitamin D, serum phosphorus, serum uric acid, PIR, education level, smoking and drinking behaviors, high pressure, diabetes, physical activities, and statin use. Then, the continuous variable serum RC was changed to a categorical variable (quartiles) to detect the correlation of RC with BMD. Stratified analyses and interaction tests were performed by sex (men and women), age (< 50 and ≥ 50), race (Other Hispanic, Non-Hispanic Black, Mexican American, Other Race, and Non-Hispanic White), and BMI (< 25, 25–30, > 30). To explore the effect of menopause on this relationship, further stratified analyses of menopausal women (premenopausal women and postmenopausal women) were conducted. Menopause was defined as the absence of menstruation in the past year because of hysterectomy or natural menopause/life change reasons [23]. The nonlinear association between RC and total spine BMD was detected by restricted cubic spline (RCS). All analyses were conducted by EmpowerStats (4.0) and R software (4.3.1) using MEC weight. A *P* value < 0.05 was deemed to indicate statistical significance.

## Results

### Baseline characteristics

A sample of 3815 subjects ≥ 20 years old were recruited for the present analyses; 1905 (49.93%) were men, and 1910 (50.07%) were women, with an average age of 39.78 ± 11.51 years. In the final analyses, 34.36% were Non-Hispanic White, 20.73% were Non-Hispanic Black, 18.77% were Other Race, 15.20% were Mexican American, and 10.93% were Other Hispanic. The mean serum RC of participants was 0.56 mmol/L. Individuals with higher RC were more likely to be women, older, Non-Hispanic White, Mexican American, less educated, poor, frequent smokers, diabetic, and hypertensive. They used more statins and had higher BMIs; serum uric acid, serum calcium, blood urea nitrogen, and serum vitamin D levels; and lower total spine BMD (Table 1).

**Table 1:** Characteristics of the study population based on RC quartiles

		Remnant cholesterol (mmol/L)				
	total	Q1 (≤ 0.308)	Q2 (0.309–0.464)	Q3 (0.465–0.699)	Q4 (≥ 0.700)	*P* value
Number of subjects (n)	3815	933	965	959	958	
Age (years)	39.78 ± 11.51	36.34 ± 11.21	38.94 ± 11.73	41.37 ± 11.43	42.36 ± 10.70	< 0.001
Sex, n (%)						< 0.001
Men	1905 (49.93%)	442 (45.80%)	491 (51.20%)	583 (60.86%)	389 (41.69%)	
Women	1910 (50.07%)	523 (54.20%)	468 (48.80%)	375 (39.14%)	544 (58.31%)	
Race/ethnicity, n (%)						< 0.001
Mexican American	580 (15.20%)	91 (9.75%)	140 (14.51%)	163 (17.00%)	186 (19.42%)	
Other Hispanic	417 (10.93%)	77 (8.25%)	94 (9.74%)	127 (13.24%)	119 (12.42%)	
Non-Hispanic White	1311 (34.36%)	312 (33.44%)	304 (31.50%)	335 (34.93%)	360 (37.58%)	
Non-Hispanic Black	791 (20.73%)	289 (30.98%)	241 (24.97%)	165 (17.21%)	96 (10.02%)	
Other Race	716 (18.77%)	186 (19.27%)	169 (17.62%)	197 (20.56%)	164 (17.58%)	
BMI	29.15 ± 7.12	26.70 ± 6.72	28.80 ± 7.12	29.99 ± 7.23	31.06 ± 6.68	< 0.001
PIR	2.50 ± 1.57	2.54 ± 1.54	2.49 ± 1.60	2.50 ± 1.58	2.46 ± 1.56	0.630
Education, n (%)						< 0.001
< High school diploma	717 (18.79%)	136 (14.58%)	153 (15.85%)	199 (20.75%)	229 (23.90%)	
High school diploma	838 (21.97%)	193 (20.69%)	223 (23.11%)	216 (22.52%)	206 (21.50%)	
≥Some college	2260 (59.24%)	604 (64.74%)	589 (61.04%)	544 (56.73%)	523 (54.59%)	
Blood urea nitrogen (mmol/L)	4.58 ± 1.63	4.45 ± 1.36	4.52 ± 1.69	4.63 ± 1.74	4.72 ± 1.68	0.002
Serum calcium (mmol/L)	2.33 ± 0.08	2.32 ± 0.08	2.33 ± 0.08	2.32 ± 0.08	2.33 ± 0.08	0.038
Serum phosphorus (mmol/L)	1.18 ± 0.18	1.18 ± 0.17	1.18 ± 0.18	1.17 ± 0.18	1.17 ± 0.18	0.443
Total protein (g/L)	71.71 ± 4.27	71.47 ± 4.26	71.65 ± 4.13	71.74 ± 4.35	71.97 ± 4.32	0.087
Serum uric acid (umol/L)	319.95 ± 83.24	287.26 ± 71.86	309.38 ± 75.82	330.06 ± 81.30	352.31 ± 88.72	< 0.001
Serum vitamin D (nmol/L)	61.02 ± 25.18	59.67 ± 25.60	59.67 ± 24.31	61.99 ± 25.65	62.73 ± 25.05	0.010
4/5 or more drinks every day, n (%)						0.072
Yes	477 (12.50%)	95 (10.18%)	127 (13.16%)	128 (13.35%)	127 (13.26%)	
No	2647 (69.38%))	668 (71.60%)	644 (66.74%)	658 (68.61%)	677 (70.67%)	
No reported	691 (18.11%)	170 (18.22%)	194 (20.10%)	173 (18.04%)	154 (16.08%)	
Smoked at least 100 cigarettes in life, n (%)						< 0.001
Yes	1524 (39.95%)	298 (31.94%)	361 (37.41%)	397 (41.40%)	468 (48.85%)	
No	2291 (60.05%)	635 (68.06%)	604 (62.59%)	562 (58.60%)	490 (51.15%)	
Every told you had high blood pressure, n (%)						< 0.001
Yes	945 (24.77%)	147 (15.76%)	235 (24.35%)	250 (26.07%)	313 (32.67%)	
No	2870 (75.23%)	786 (84.24%)	730 (75.65%)	709 (73.93%)	645 (67.33%)	
Doctor told you had high blood diabetes, n (%)						< 0.001
Yes	281 (7.37%)	25 (2.68%)	55 (5.70%)	74 (7.72%)	127 (13.26%)	
No	3449 (90.41%)	892 (95.61%)	891 (92.33%)	854 (89.05%)	812 (84.76%)	
Borderline	85 (2.23%)	16 (1.71%)	19 (1.97%)	31 (3.23%)	19 (1.98%)	
Days moderate recreational activities in a typical week, n (%)						< 0.001
≤ 3	1054 (27.63%)	285 (30.55%)	271 (28.08%)	257 (26.80%)	241 (25.16%)	
≥ 4	627 (16.44%)	192 (20.58%)	173 (17.93%)	146 (15.22%)	116 (12.11%)	
No reported	2134 (55.94%)	456 (48.87%)	521 (53.99%)	556 (57.98%)	601 (62.73%)	
Statin use, n (%)						< 0.001
Yes	545 (14.29%)	47 (5.04%)	109 (11.30%)	163 (17.00%)	226 (23.59%)	
No	3270 (85.71%)	886 (94.96%)	856 (88.70%)	796 (83.00%)	732 (76.41%)	
Total spine BMD (g/cm^2^)	1.03 ± 0.15	1.05 ± 0.15	1.04 ± 0.15	1.03 ± 0.16	1.00 ± 0.14	< 0.001
Remnant cholesterol (mmol/L)	0.56 ± 0.43	0.23 ± 0.05	0.38 ± 0.05	0.57 ± 0.07	1.06 ± 0.57	< 0.001

Mean ± SD for continuous variables: the *P* value was calculated by the weighted linear regression model. (%) for categorical variables. The *P* value was calculated by the weighted chi-square test. Abbreviation: BMI: body mass index. PIR: poverty income ratio. BMD: bone mineral density. RC: remnant cholesterol.

### Associations of serum RC with total spine BMD

**Fig. 2:**
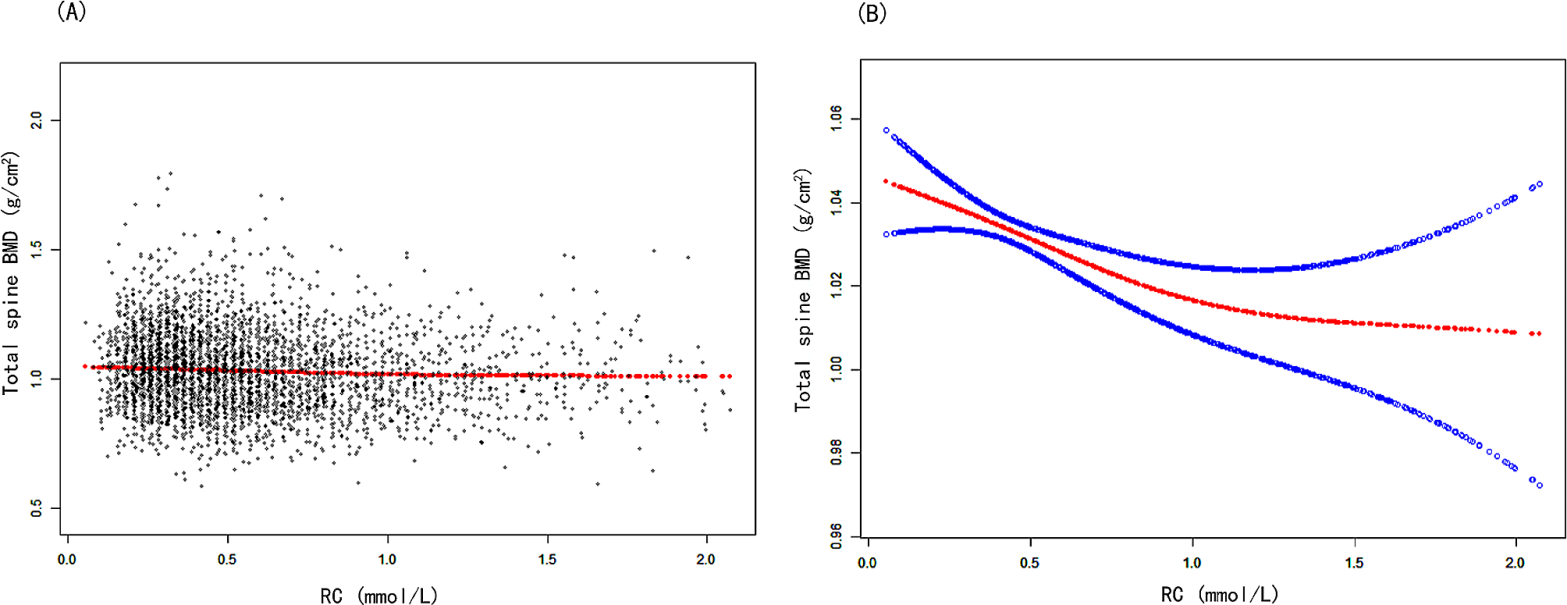
The association between poverty RC and total spine BMD. **(A)** Each black point represents a sample. **(B)** Red line represents the smooth curve fit between variables. Blue lines represent the 95% of confidence interval from the fit. Age, sex, race, PIR, education level, BMI, total protein, blood urea nitrogen, serum total calcium, serum phosphorus, serum uric acid, serum vitamin D, smoking and drinking behaviors, high pressure, diabetes, physical activities, and statin use were adjusted. Abbreviation: PIR: poverty income ratio. BMI: body mass index. BMD: bone mineral density. RC: remnant cholesterol

**Fig. 3:**
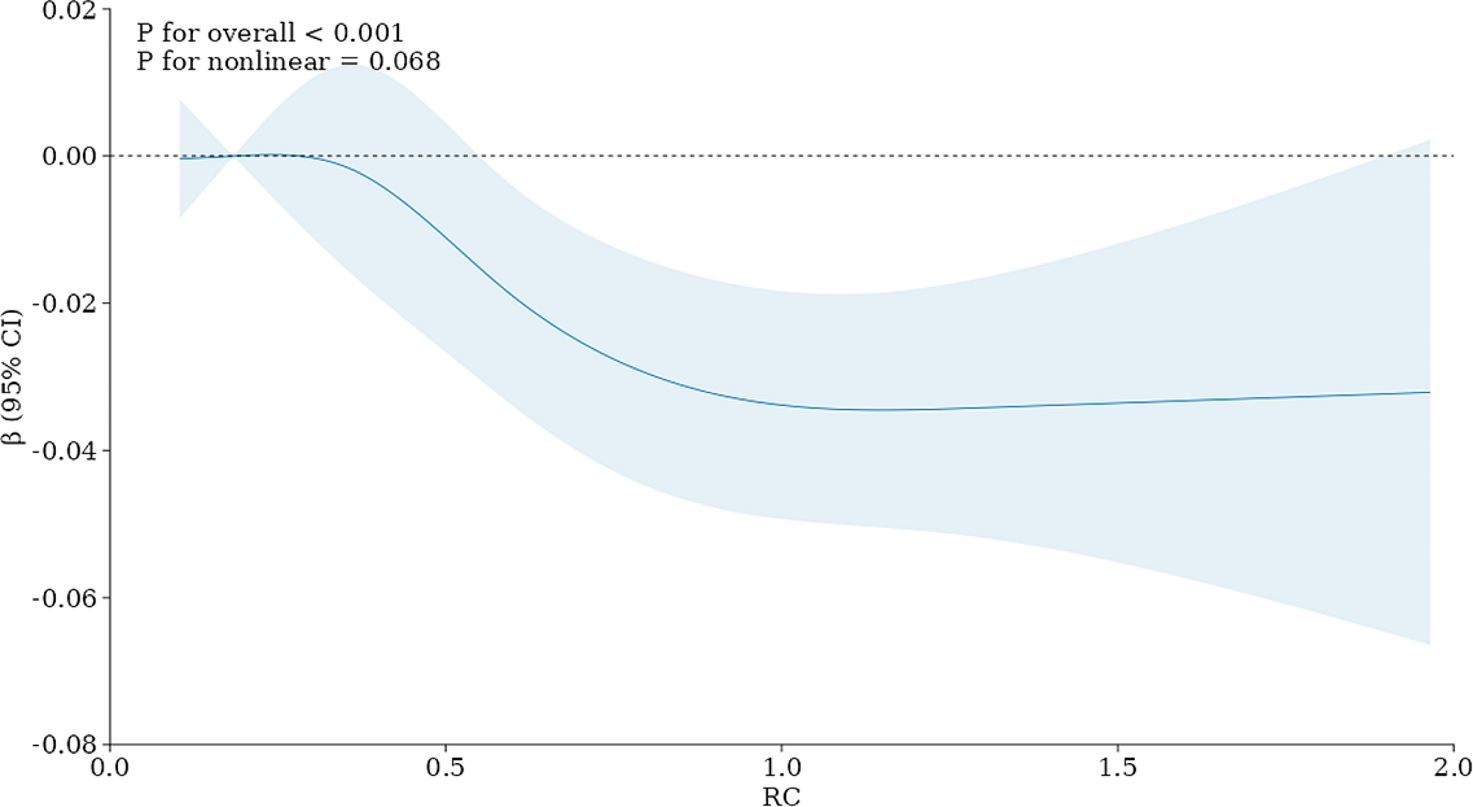
Restricted cubic spline (RCS) analysis with multivariate-adjusted associations between RC and total spine BMD. Age, sex, race, PIR, education level, BMI, total protein, blood urea nitrogen, serum total calcium, serum phosphorus, serum uric acid, serum vitamin D, smoking and drinking behaviors, high pressure, diabetes, physical activities, and statin use were adjusted. Abbreviation: PIR: poverty income ratio. BMI: body mass index. BMD: bone mineral density. RC: remnant cholesterol

Models 1 and 2 revealed a negative association of RC with total spine BMD (Table 2). In Model 3, a negative correlation was also observed between serum RC and total spine BMD (β= -0.024, 95% CI: -0.039, -0.010). Figure 2 showed the smooth curve fittings of the correlation of serum RC with total spine BMD. Then, the continuous variable of RC was changed to a categorical variable (quartiles). According to Model 3, subjects in the highest quarter of serum RC have a significantly reduced BMD compared to subjects in the first quarter. (β= -0.024, 95% CI: -0.038, -0.010). Figure 3 showed the results of RCS. Consistent with the findings of the multivariate linear regression analyses, the dose–response association also indicated a negative linear association of serum RC with total spine BMD (nonlinear *P* = 0.068, overall *P* < 0.001) (Fig. 3).

### Stratified analysis and interaction testing

Stratified analysis and interaction tests were conducted for age, sex, race, and BMI (Table 2). The interaction tests showed that those subgroups had no significant effect on the relationship (*P* for interaction > 0.05). In Model 3, the results revealed a negative correlation of RC with total spine BMD in men (β= − 0.030, 95% CI: − 0.050, − 0.011) but not in women (β= − 0.011, 95% CI: − 0.034, 0.012). However, no interaction effect was found (*P* for interaction = 0.173). For race, the results found the negative association was stronger in Non-Hispanic White (β= − 0.030, 95% CI: − 0.053, − 0.007) than others. Nevertheless, the results found no interaction effect (*P* for interaction = 0.767). For age groups, the results showed a negative association of RC with total spine BMD in individuals aged < 50 years (β= − 0.033, 95% CI: − 0.049, − 0.016) but not in individuals aged ≥ 50 years (β= − 0.010, 95% CI: − 0.039, 0.019). (*P* for interaction = 0.148). For all three BMI groups, the results indicated a negative correlation of serum RC with total spine BMD: BMI < 25: (β= -0.030, 95% CI: -0.042, -0.018); 25 ≤ BMI ≤ 30: (β= -0.033, 95% CI: -0.057, -0.010); BMI > 30: (β= -0.021, 95% CI: -0.044, -0.003). Further stratified analyses were conducted by menopause status in women (Appendix 1). The results showed that menopause status has no interaction effect on the correlation of serum RC with total spine BMD in women (*P* for interaction = 0.582).

**Table 2:** The association between serum RC and total spine bone mineral density (g/cm^2^)

	Model 1 β (95% CI) *P* value	Model 2 β (95% CI) *P* value	Model 3 β (95% CI) *P* value
RC	-0.044 (-0.058, -0.030) < 0.001	-0.019 (-0.033, -0.005) 0.007	-0.024 (-0.039, -0.010) < 0.001
RC categories			
Q1	Reference	Reference	Reference
Q2	-0.008 (-0.022, 0.005) 0.232	0.002 (-0.011, 0.015) 0.767	-0.001 (-0.014, 0.013) 0.997
Q3	-0.025 (-0.038, -0.011) < 0.001	-0.005 (-0.018, 0.009) 0.496	-0.008 (-0.022, 0.006) 0.249
Q4	-0.045 (-0.059, -0.031) < 0.001	-0.018 (-0.031, -0.004) 0.011	-0.024 (-0.038, -0.010) 0.001
P for trend	< 0.001	0.007	< 0.001
Subgroup analysis stratified by sex			
Men	-0.045 (-0.063, -0.026) < 0.001	-0.028 (-0.046, -0.010) 0.002	-0.030 (-0.050, -0.011) < 0.001
Women	-0.049 (-0.070, -0.027) < 0.001	-0.019 (-0.042, 0.003) 0.097	-0.011 (-0.034, 0.012) 0.351
P for interaction	0.741	0.135	0.173
Stratified analysis by race/ethnicity			
Mexican American	-0.029 (-0.060, 0.002) 0.066	-0.022 (-0.054, 0.009) 0.168	-0.018 (-0.050, 0.013) 0.256
Other Hispanic	-0.030 (-0.071, 0.012) 0.159	-0.024 (-0.066, 0.019) 0.274	-0.019 (-0.064, 0.026) 0.402
Non-Hispanic White	-0.030 (-0.051, -0.009) 0.005	-0.024 (-0.045, -0.002) 0.033	-0.030 (-0.053, -0.007) 0.010
Non-Hispanic Black	-0.005 (-0.024, 0.015) 0.650	-0.002 (-0.022, 0.017) 0.810	-0.012 (-0.059, 0.036) 0.624
Other races (Including multi-racial)	-0.025 (-0.053, 0.002) 0.074	-0.023 (-0.052, 0.006) 0.117	-0.030 (-0.061, 0.001) 0.056
P for interaction	0.324	0.533	0.767
Stratified analysis by age			
< 50	-0.029 (-0.041, -0.016) < 0.001	-0.026 (-0.042, -0.010) 0.001	-0.033 (-0.049, -0.016) < 0.001
≥ 50	-0.010 (-0.037, 0.016) 0.438	0.004 (-0.023, 0.031) 0.787	-0.010 (-0.039, 0.019) 0.484
P for interaction	0.212	0.025	0.148
Stratified analysis by BMI			
< 25	-0.086 (-0.173, 0.000) 0.053	-0.045 (-0.078, -0.012) 0.007	-0.030 (-0.042, -0.018) < 0.001
25–30	-0.045 (-0.078, -0.012) 0.007	-0.009 (-0.042, 0.024) 0.580	-0.033 (-0.057, -0.010) 0.008
> 30	-0.031 (-0.044, -0.019) < 0.001	-0.032 (-0.067, 0.002) 0.068	-0.021 (-0.044, -0.003) 0.040
P for interaction	0.439	0.692	0.377

Model 1 adjusted no covariates. Model 2 adjusted age, sex, and race. Model 3 adjusted age, sex, race, PIR, education level, BMI, total protein, blood urea nitrogen, serum total calcium, serum phosphorus, serum uric acid, serum vitamin D, smoking and drinking behaviors, high pressure, diabetes, physical activities, and statin use. Abbreviation: BMI: body mass index. PIR: poverty income ratio. BMD: bone mineral density. RC: remnant cholesterol.

### The effect of TC, LDL-C, HDL-C, and RC on total spine BMD

The effects of RC and classical lipid composition on total spine BMD were compared (Table 3). RC had a greater impact on BMD than did TC, LDL-C, or HDL-C. According to the fully adjusted model, every unit increase in TC, LDL-C, and RC was related to decreases in total spine BMD of 0.010, 0.013, and 0.024 g/cm^2^, respectively. Every unit increase in HDL-C was related to a 0.022 g/cm^2^ increase in total spine BMD.

**Table 3:** The effect of TC, LDL-C, HDL-C, and RC on total spine BMD (g/cm^2^)

Exposure	Model 1 β (95% CI) *P* value	Model 2 β (95% CI) *P* value	Model 3 β (95% CI) *P* value
TC	-0.017 (-0.021, -0.012) < 0.001	-0.011 (-0.016, -0.007) < 0.001	-0.010 (-0.015, -0.005) < 0.001
LDL-C	-0.018 (-0.024, -0.013) < 0.001	-0.014 (-0.019, -0.009) < 0.001	-0.013 (-0.018, -0.007) < 0.001
HDL-C	0.020 (0.008, 0.032) 0.001	0.014 (0.002, 0.027) 0.023	0.022 (0.009, 0.035) 0.002
RC	-0.044 (-0.058, -0.030) < 0.001	-0.019 (-0.033, -0.005) 0.007	-0.024 (-0.039, -0.010) < 0.001

Model 1 adjusted no covariates. Model 2 adjusted age, sex, and race. Model 3 adjusted age, sex, race, PIR, education level, BMI, total protein, blood urea nitrogen, serum total calcium, serum phosphorus, serum uric acid, serum vitamin D, smoking and drinking behaviors, high pressure, diabetes, physical activities, and statin use. Abbreviation: BMD: bone mineral density. TC: total cholesterol. LDL-C: low-density lipoprotein cholesterol. HDL-C: high-density lipoprotein cholesterol. RC: remnant cholesterol. BMI: body mass index. PIR: poverty income ratio.

## Discussion

This study first explored the correlation of serum RC with BMD in U.S. adults. The results showed a negative correlation of serum RC with total spine BMD in Americans aged ≥ 20 years old. The interaction tests showed that the age, sex, race, and BMI subgroups had no significant effect on the relationship. Dose-response analysis by RCS also confirmed a negative linear relationship between serum RC and BMD. These findings emphasize the critical role of RC in bone health.

RC includes cholesterol in very low-density lipoproteins (VLDLs), cholesterol in celiac residue in the postprandial state, and intermediate-density lipoproteins (IDLs) in the fasting state. Many recent NHANES studies have identified RC as a biomarker of various diseases [24–27]. In a survey of 7777 subjects from the NHANES 1999–2008, Yan et al. [24] reported a positive association between serum RC and rheumatoid arthritis (RA). Thus, RC may serve as an essential predictor of RA occurrence. Zhang et al. [25] found that RC was positively associated with cardiovascular mortality in a cohort of 19,650 individuals from the NHANES 1999–2014. This positive association was independent of traditional risk factors like HDL-C and LDL-C. He et al. [26] established a link between RC and albuminuria or renal function. They found a negative correlation between serum RC and eGFR (β= -2.12, 95% CI: -3.04, -1.21). Xie et al. [19] observed that serum RC was negatively associated with memory function and verbal learning in individuals aged ≥ 60 years. Therefore, lower levels of RC may be beneficial for preventing cognitive impairment in older adults. In another study by Chen et al. [27], a nonlinear association between RC and nonalcoholic fatty liver disease (NAFLD) was found. Risk ratios for NAFLD on the left and right sides of the inflection point are 3.88 (95% CI: 2.43–6.2) and 0.59 (95% CI: 0.21–1.71). Nevertheless, the possible link between RC and BMD has not been well explained. Hou et al. [18] investigated the correlation of RC with BMD in 7053 Chinese men, and they indicated that serum RC was negatively related to hip BMD. Every one mmol/l increase in RC was related to a 0.0079 g/cm^2^ decrease in hip BMD. Furthermore, compared with people of the lowest serum RC quartile, people of the highest serum RC quartile have a 1.43-fold risk of low bone mass. However, the subjects in this study were only middle-aged men. It is well known that advanced age and women are high-risk factors for osteoporosis. Therefore, the correlation of RC with BMD in older people and women is still unknown. This study first indicated a negative correlation of RC with BMD in Americans ≥ 20 years. Furthermore, the interaction tests showed that age and sex have no interaction effects on the final results (*P* for interaction > 0.05). Thus, RC may serve as a novel lipid metabolism marker to predict bone health in the population.

Although no studies have investigated the mechanisms of RC in bone metabolism, many basic investigations have assessed the impacts of classical lipid metabolism on bone health. Overall, the effect of abnormal lipid metabolism on bone health is complex and involves several factors. Both osteoblasts and adipocytes are derived from the differentiation of mesenchymal stem cells (MSCs) [28]. The Wnt/β-catenin signalling pathway regulates this differentiation process [29]. When this pathway is blocked, MSCs will favour adipocyte differentiation. In a study by Pelton et al. [30], after four months of feeding, mice fed a high-cholesterol diet showed significantly reduced femoral and cranial BMD compared to those fed a low- or no-cholesterol diet. They also suggested that high cholesterol intake could inhibit osteoblast differentiation and enhance osteoclast function in mice [30]. Furthermore, high TC and LDL-C can increase bone marrow microcirculatory stress and decrease bone vascularization, leading to bone loss [31].

Epidemiological studies have also widely discussed the relationship of lipid profiles with BMD. Most studies showed a negative or no correlation of LDL-C and TC with BMD [7, 32, 33]. The adverse effects of LDL-C and TC on bone health can often be attributed to vascular calcification and atherosclerosis [34, 35]. However, studies showed a positive, negative, or no correlation between HDL-C and BMD [36–39]. In a study by Sun et al. [38], their survey indicated that HDL-C was negatively related to lumbar BMD after adjusting a large range of confounders. However, Zolfaroli et al. [39] found a positive correlation of HDL-C with total spine and femur neck BMD in postmenopausal females. These differences may be explained in part by differences in study population, study methodology, or error control. In total, the relationship between traditional lipid profiles and BMD has been controversial in previous studies. This study identified a negative association of serum RC, TC, and LDL-C with total spine BMD in U.S. adults, with the effect of RC being stronger. In contrast, HDL-C was found to have a positive association. Thus, RC could be a new predictor of low BMD in clinical practice.

### Strengths and limitations

The study has a variety of strengths. First, this study first assessed the correlation of RC with BMD in U.S. adults. The study used data from the recent cycles in the NHANES 2013–2018, which represent the general American population. Second, this study suggested that RC may serve as a new bone health marker, revealing the importance of lipid metabolism disorders in the mechanisms of bone loss. Third, linear regression analyses were conducted after adjusting for a large number of confounders. In addition, the nonlinear RCS test made the results more reliable.

The present study also has several limitations. First, some confounders in this study were collected through questionnaires and recall, which may suffer from recall bias and lead to inaccuracies. Second, although the study adjusted for many confounders, there remains the possibility of residual confounding. Third, the results cannot be used to make causal inferences because of the nature of cross-sectional studies. Further prospective studies are required to validate their causal inferences.

## Conclusions

This study indicated a negative correlation of serum RC with total spine BMD in U.S. adults. Moreover, RC showed a stronger effect on total spine BMD than TC, LDL-C, and HDL-C. The finding emphasized the important role of RC in bone health in Americans. RC may be a new indicator for the early detection and prevention of osteoporosis.

### Electronic supplementary material

Below is the link to the electronic supplementary material.

Supplementary Material 1. **Appendix 1**: stratified analysis by menopause status in women

Supplementary Material 2

Supplementary Material 3

## Data Availability

The datasets generated and/or analysed during the current study are available in the [NHANES] repository, [https://www.cdc.gov/nchs/nhanes/].

## Abbreviations

RC: Remnant cholesterol
BMD: Bone mineral density
RCS: Restricted cubic spline
NHANES: National Health and Nutrition Examination Survey
LDL-C: Low-density lipoprotein cholesterol
TC: Total cholesterol
HDL-C: High-density lipoprotein cholesterol
PBM: Peak bone mass
PIR: Poverty income ratio
BMI: Body mass index
VLDLs: Very low-density lipoproteins
IDLs: Intermediate-density lipoproteins
RA: Rheumatoid arthritis
NAFLD: Nonalcoholic fatty liver disease
MSCs: Mesenchymal stem cells
