# Supplementary material for: The association between remnant cholesterol and bone mineral density in US adults: the National Health and Nutrition Examination Survey (NHANES) 2013–2018
Source: Lipids Health Dis. 2024 May 18;23:148. doi: 10.1186/s12944-024-02145-6 (PMC11102129; doi:10.1186/s12944-024-02145-6)
Supplement: Supplementary file 1 — Supplementary Material 1. Appendix 1: stratified analysis by menopause status in women [file 12944_2024_2145_MOESM1_ESM.docx]

**Appendix 1**: Stratified analysis by menopause status in women.

|  | Model 1 β (95% CI) *P* value | Model 2 β (95% CI) *P* value | Model 3 β (95% CI) *P* value |
| --- | --- | --- | --- |
| Premenopausal women | -0.035 (-0.060, -0.010) 0.006 | -0.009 (-0.035, 0.016) 0.466 | -0.010 (-0.036, 0.017) 0.474 |
| Postmenopausal women | -0.014 (-0.054, 0.026) 0.507 | 0.002 (-0.038, 0.041) 0.925 | -0.012 (-0.057, 0.033) 0.592 |
| P for interaction | 0.350 | 0.620 | 0.582 |

N=1910. Model 1 adjusted no covariates. Model 2 adjusted age and race. Model 3 adjusted age, race, PIR, education level, BMI, total protein, blood urea nitrogen,

serum total calcium, serum phosphorus, serum uric acid, serum vitamin D, smoking and drinking behaviors, high pressure, diabetes, physical activities, and statin use.

Abbreviation: BMI: body mass index. PIR: poverty income ratio.
